# Chemogenetic inhibition of central amygdala CRF-expressing neurons decreases alcohol intake but not trauma-related behaviors in a rat model of post-traumatic stress and alcohol use disorder

**DOI:** 10.1038/s41380-024-02514-8

**Published:** 2024-03-21

**Authors:** Bryan Cruz, Valentina Vozella, Vittoria Borgonetti, Ryan Bullard, Paula C. Bianchi, Dean Kirson, Luisa B. Bertotto, Michal Bajo, Roman Vlkolinsky, Robert O. Messing, Eric P. Zorrilla, Marisa Roberto

**Affiliations:** 1 Department of Molecular Medicine, The Scripps Research Institute, La Jolla, CA 92073 USA; 2 Department of Pharmacology, Addiction Science, and Toxicology, The University of Tennessee Health Science Center, Memphis, TN 38163 USA; 3 Waggoner Center for Alcohol and Addiction Research, Department of Neuroscience, The University of Texas at Austin, Austin, TX 78712 USA

**Keywords:** Neuroscience, Physiology, Psychology

## Abstract

Post-traumatic stress disorder (PTSD) and alcohol use disorder (AUD) are often comorbid. Few treatments exist to reduce comorbid PTSD/AUD. Elucidating the mechanisms underlying their comorbidity could reveal new avenues for therapy. Here, we employed a model of comorbid PTSD/AUD, in which rats were subjected to a stressful shock in a familiar context followed by alcohol drinking. We then examined fear overgeneralization and irritability in these rats. Familiar context stress elevated drinking, increased fear overgeneralization, increased alcohol-related aggressive signs, and elevated peripheral stress hormones. We then examined transcripts of stress- and fear-relevant genes in the central amygdala (CeA), a locus that regulates stress-mediated alcohol drinking. Compared with unstressed rats, stressed rats exhibited increases in CeA transcripts for *Crh* and *Fkbp5* and decreases in transcripts for *Bdnf* and *Il18*. Levels of *Nr3c1* mRNA, which encodes the glucocorticoid receptor, increased in stressed males but decreased in stressed females. Transcripts of *Il18* binding protein (*Il18bp*), *Glp-1r*, and genes associated with calcitonin gene-related peptide signaling (*Calca*, *Ramp1*, *Crlr-1*, and *Iapp)* were unaltered. *Crh*, but not *Crhr1*, mRNA was increased by stress; thus, we tested whether inhibiting CeA neurons that express corticotropin-releasing factor (CRF) suppress PTSD/AUD-like behaviors. We used *Crh-*Cre rats that had received a Cre-dependent vector encoding hM4D(Gi), an inhibitory Designer Receptors Exclusively Activated by Designer Drugs. Chemogenetic inhibition of CeA CRF neurons reduced alcohol intake but not fear overgeneralization or irritability-like behaviors. Our findings suggest that CeA CRF modulates PTSD/AUD comorbidity, and inhibiting CRF neural activity is primarily associated with reducing alcohol drinking but not trauma-related behaviors that are associated with PTSD/AUD.

## Introduction

Post-traumatic stress disorder (PTSD) commonly is comorbid with substance use disorders, including alcohol use disorder (AUD) [1–3]. Symptoms of comorbid PTSD/AUD include excessive generalized fear and anxiety, drinking-related aggression, and alcohol drinking to cope with emotional dysregulation (i.e., negative reinforcement) [4–7]. Individuals with PTSD also develop AUD more rapidly than non-PTSD individuals, with heightened negative affective phenotypes, such as suicidal ideation and social isolation [6, 8, 9]. Pharmacotherapies to treat both PTSD/AUD remain limited. Thus, identifying shared neural mechanisms of PTSD/AUD may reveal new therapeutic avenues to treat these comorbid disorders.

Stress-mediated alcohol drinking is influenced by neuroendocrine stress responses via the hypothalamic-pituitary-adrenal (HPA) axis [10–12]. Stress canonically activates the HPA axis via the release of corticotropin-releasing factor (CRF) into portal blood, and it also orchestrates an array of additional behavioral and physiological responses to stress and alcohol [13] via the paracrine/autocrine release of diverse peptides into non-neuroendocrine circuits. One such region, the central amygdala (CeA), contains extrahypothalamic CRF neurons that release CRF locally within and outside the CeA, and this CeA CRF system can modulate stress and alcohol drinking in rodents [13, 14]. In animal models, CRF is released in the CeA during alcohol withdrawal [15], and CRF_1_ receptor blockade decreases escalated drinking [16–18] and negative affective behaviors that are associated with alcohol withdrawal [15, 19]. Importantly, the inhibition of CeA CRF-expressing neurons reduces escalated alcohol drinking and physical signs of alcohol withdrawal in rats [20].

Much literature has broadened our understanding of the involvement of CeA CRF in stress and alcohol drinking. Newly emerging studies have provided new insights into other neuropeptides and proteins in the CeA that are also regulated by stress and alcohol. Recent work demonstrated that stress and alcohol drinking influence glucocorticoid receptor (GR) signaling, increasing the abundance of FK-binding protein 51 (FKBP5), a co-chaperone that is key for GR transcriptional activity [21–25]. Another neuropeptide that is influenced by stress and alcohol is calcitonin gene-related peptide (CGRP). CGRP immunofluorescence increases in cortical and limbic brain regions following alcohol exposure and withdrawal, and CGRP+ neurons from the parabrachial nucleus synapse onto CeA neurons [26, 27]. Furthermore, the role of neuroinflammatory signaling has also gained interest in stress-mediated alcohol drinking. Alcohol intoxication increases interleukin-18 (IL-18). Recent work from our group found that IL-18 signaling in the CeA is impaired after traumatic stress and alcohol drinking [28–30]. Thus, it is important to understand whether these molecular systems play a role in PTSD/AUD.

We recently developed a comorbid model of PTSD/AUD-like behavior [31]. This is an avoidance-based model of traumatic stress that combines re-experiencing footshock in a familiar context with alcohol drinking. This procedure results in fear overgeneralization and irritability-like behaviors post-alcohol abstinence and greater alcohol drinking and anxiety-like behavior that resemble symptoms of comorbid PTSD/AUD [31, 32]. The present study employed this model to compare effects of familiar “2-hit” stress *vs*. no stress in male and female rats. We then assessed CeA expression of stress-related genes, including CRF (C*rh)*, CRF_1_ receptor (C*rhr1)*, *Fkbp5*, brain-derived neurotrophic factor (*Bdnf*), and nuclear receptor subfamily 3 group C member 1 (*Nr3c1*; which encodes the GR), which are known to modulate anxiety and alcohol drinking [23, 33]. We also evaluated transcripts for CGRP system-related gene targets, such as calcitonin-related polypeptide α (*Calca*), receptor activity modify protein 1 (*Ramp1*), calcitonin receptor-like receptor 1 (*Crlr-1*), glucagon-like peptide 1 (*Glp-1r*), and islet amyloid polypeptide (*Iapp*), and neuroinflammatory targets, including *Il18* and *Il18* binding protein (*Il18bp*) [30, 34–37]. Among the transcript changes, we observed a significant increase in *Crh*, but not *Crhr1*, mRNA induced by stress, thus, we tested whether chemogenetic inhibition of CeA CRF-expressing neurons modulates phenotypes of comorbid PTSD/AUD in *Crh*-Cre male and female rats and examined sex differences. The overall aim of targeting CRF is to provide deeper insight into CeA CRF pro-stress role in modulating PTSD associated alcohol drinking.

## Methods and materials

### Animals

A total of *n* = 71 wildtype Wistar and *n* = 50 heterozygous *Crh-*Cre (Wistar background) male and female rats were used in this study [38]. All rats were pair-housed, separated by a perforated clear Plexiglas divider, and had access to food and water *ad libitum* before the experiments began. All experimental procedures were approved by The Scripps Research Institute (TSRI) Institutional Animal Care and Use Committee (protocol no. 09-0006). All procedures followed the National Institutes of Health *Guide for the Care and Use of Laboratory Animals* (8th edition).

### Overall approach

This report consisted of three studies that were conducted in separate cohorts of rats. **Study 1** (*n* = 35 rats) examined effects of stress and alcohol in an established paradigm of comorbid PTSD/AUD [31, 32]. This study utilized familiar inhibitory avoidance shock stress procedures, followed by alcohol drinking, fear overgeneralization, bottle bush irritability, and assessments of peripheral stress hormones (corticosterone [CORT] and adrenocorticotropic hormone [ACTH]). Unstressed controls underwent similar procedures, except they did not receive footshocks. To understand the role of other stress-related peptide genes, **Study 2** (*n* = 36 rats) examined transcript levels for *Crh*, *Crhr1*, *Fkbp5*, *Bdnf*, *Nr3c1*, *Calca*, *Il18*, *Il18bp*, *Ramp1*, *Crlr-1*, *Iapp* (which encodes pro-amylin*)*, and *Glp-1r* in a separate cohort of rats that underwent a similar comorbid PTSD/AUD 2-hit model procedure. A separate cohort of rats (*n* = 25) was tested for changes in these genes under shock stress but non-drinking conditions. In **Study 3** (*n* = 50), CeA CRF-expressing neurons in *Crh-*Cre rats were transduced for Cre-dependent Designer Receptors Exclusively Activated by Designer Drugs (DREADDs) or control vector (Cre-dependent) to selectively inhibit these CRF neurons. Surgeries occurred after footshock stress. The rats were allowed to recover from surgery and then received access to alcohol drinking, followed by testing for fear overgeneralization and irritability-like behavior. The administration of clozapine-*N*-oxide (CNO; 2 mg/kg, i.p.) or vehicle (5% dimethylsulfoxide [DMSO] with 0.9% saline) was given on the last day of drinking and on each behavioral test day.

### Familiar footshock stress

The “2-hit” footshock stress procedures were conducted using a familiar context in an inhibitory avoidance procedure as previously reported [31, 32, 39]. Footshock stress (3.0 mA for 2 s) was administered on two single shock instances after crossing from a lit compartment to a dark compartment in the same contextual environment (familiar), separated by 48 h. The latency to cross (in seconds) into the dark compartment was measured. Unstressed controls underwent similar procedures without the presence of footshock.

### Voluntary alcohol regimen

Two weeks after the initial shock, all rats received an initial 48 h acclimation to the alcohol (20% *v/v*) bottle in addition to the water bottle, followed by intermittent (Monday, Wednesday, Friday), two-bottle choice (2BC) limited access (2 h) for 4 weeks in their home cage as previously described [31, 32, 39]. Blood alcohol levels (BALs) were measured in the middle of week 4 (Day 11 of 2BC), 30 min into their 2 h session before rats began their abstinence period. To validate BALs, intake and preference in rats that showed detectable levels within the 30 min were compared to rats that displayed non-detectable levels (see Fig. S1a, b) as previously reported [39].

### Fear overgeneralization

All rats were tested in a fear overgeneralization paradigm that consisted of a modified inhibitory avoidance box and were given a maximum of 10 min to enter the dark compartment as previously described [31, 32, 39]. The latency to cross (in seconds) into the dark compartment was recorded, and animals that never crossed were recorded as having a latency of 600 s (10 min).

### Bottle-brush irritability

Bottle-brush irritability was assessed by scoring aggressive-like behaviors (biting, boxing, following, and mounting), defensive behaviors (startling, digging, freezing, climbing cage walls, vocalizing, and attempting to escape), and general explorative behaviors (grooming, rearing, and exploring) as previously described [31, 39].

### Stress hormone analysis

Plasma samples were analyzed using commercially available MilliPlex kits (Millipore Sigma) that are specific for CORT, ACTH, and melatonin as previously reported [39]. Trunk blood was collected in **Study 1** in ethylenediamine tetraacetic acid (EDTA) tubes (BD Vacutainer K2 EDTA) then placed on ice 1 week after the last behavioral test during the rats’ active phase 2 h into their dark cycle (between 10 AM and 2 PM). The centrifuged plasma samples were analyzed on a MAGPIX system using xPONENT software. The intra-assay coefficients of variation were < 10%.

### Quantitative polymerase chain reaction

Rats were anesthetized with isoflurane and rapidly decapitated. Brains were harvested, flash frozen in dry ice-cooled isopentane, and stored at −80 °C until analysis. The CeA was dissected from coronal cryostat sections (400 µm) using a stainless-steel punch. mRNA was isolated from the CeA using Trizol (Invitrogen; catalog no. 15596026) and RNA extraction kits (Zymo Research; catalog no. NC9972645). cDNA synthesis was conducted using SuperScript IV exDNAse kits (Invitrogen; catalog no. 11766050). cDNA was amplified using SYBR green PowerTrack master mix (Applied Biosystems; catalog no. A46109) and quantified by quantitative polymerase chain reaction (qPCR) on the QuantStudio 5 system. *Crh*, *Crhr1*, *Fkbp5*, *Bdnf*, *Nr3c1*, *Calca*, *Il18*, *Il18bp*, *Ramp1*, *Crlr-1*, *Iapp*, and *Glp-1r* mRNA fold changes are expressed relative to unstressed controls and were normalized to the validated and stable housekeeping gene, β-actin (*Actb*) [40]. Cycling conditions were the following: 95 °C denaturation temperature for 15 s, 60.3 °C annealing temperature for 15 s, and 72 °C extension temperature for 15 s. Primers were obtained from Integrated DNA Technologies (Coralville, IA, USA). See Table 1 for all forward and reverse primer sequences. Specificity was confirmed as a single product in the melt curve analysis.

**Table 1:** Primer sequences.

Gene	Abbreviation	Primer sequences
Corticotropin-releasing hormone	*Crh*	*Forward:* 5ʹ-TGCTCGGCTGTCCCCCAACT-3ʹ *Reverse:* 5ʹ-CTGCAGCAACACGCGGAAAAA-3ʹ
Corticotropin-releasing hormone receptor 1	*Crhr1*	*Forward:* 5ʹ-TGC CAG GAG ATT CTC AAC GAA-3ʹ *Reverse:* 5ʹ-AAA GCCGAG ATG AGG TTC CAG-3ʹ
Fk-506 binding protein 1	*Fkbp5*	*Forward:* 5ʹ-GCCGGCAAGAAACACGAGAG-3ʹ *Reverse:* 5ʹ-GAGGAGGGCCGAGTTCATT-3ʹ
Brain-derived neurotrophic factor	*Bdnf*	*Forward:* 5ʹ-GGTCGATTAGGTGGCTTCATAG-3ʹ *Reverse*: 5ʹ-CGAACAGAAACAGAGGAGAGATT-3ʹ
Nuclear receptor subfamily 2 group C member 1	*Nr3c1*	*Forward:* 5ʹ-GAAGGGAACTCCAGTCAGAAC-3ʹ *Reverse:* 5ʹ-AATGTCTGGAAGCAGTAGGTAAG-3ʹ
Calcitonin related polypeptide α	*Calca*	*Forward:* 5ʹ-CAGT CTCAGCTCCAAGTCATC-3ʹ *Reverse:* 5ʹ-TTCC AAGGTTGACCTCAAAG-3ʹ
Receptor activity modifying protein 1	*Ramp1*	*Forward:* 5ʹ-AGC CGC TTC AAA GAG GAC ATG-3ʹ *Reverse:* 5ʹ-GCC AAT CTT GTT TGC CAC GA-3ʹ
Calcitonin receptor-like receptor 1	*Crlr-1*	*Forward:* 5ʹ-AGAGCCTAAGTTGCCAACGG-3ʹ *Reverse:* 5ʹCCACTGCCGTGAGGTGAATG-3ʹ
Islet amyloid polypeptide (amylin)	*Iapp*	*Forward:* 5ʹ-ACATGTGCCACACAACGTCT-3ʹ *Reverse:* 5ʹ-ACAAACACAGCAAGCACAGG-3ʹ
Glucagon-like peptide-1 receptor	*Glp-1r*	*Forward:* 5ʹ-CCGGGTCATCTGCATCGT-3ʹ *Reverse:* 5ʹ-AGTCTGCATTTGATGTCGGTCTT-3ʹ
Interleukin 18	*Il18*	*Forward:* 5ʹ-CAAAAGAAACCCGCCTGTGT-3ʹ *Reverse:* 5ʹ-TCACAGCCAGTCCTCTTACTTCAC-3ʹ
Interleukin 18 binding protein	*Il18bp*	*Forward:* 5ʹ-ATAGTCGGGAGCTTTCCTAGA-3ʹ *Reverse:* 5ʹ-CTGGTGCCTAACTGTGACTT-3ʹ
β-actin	*Actb*	*Forward:* 5ʹ-ATCTGGCACCACACCTCC-3ʹ *Reverse:* 5ʹ-AGCCAGGTCCAGACGCA-3ʹ

Genes were expressed relative to unstressed controls and were normalized to the validated and stable housekeeping gene, β-actin.

### Stereotaxic surgery

For the chemogenetic study, rats were anesthetized with an isoflurane/oxygen mixture (1–3%) and placed on a stereotaxic apparatus. Cre-dependent viral vectors that contained the inhibitory DREADD (AAV8-hSyn-DIO-hM4D[Gi]-mCherry; titer: 1 × 10¹³ vg/ml) or reporter control (AAV8-hSyn-DIO-mCherry; titer: ≥1 × 10¹³ vg/ml) were purchased from Addgene (catalog no. 4462 and 50459). Viral vectors were injected bilaterally in the CeA (anterior/posterior: −2.16 mm; medial/lateral: ± 4.3 mm; dorsal/ventral: −8.5 mm from flat skull). The infusion rate was 150 nl/min for 5 min. After the infusion, the injectors remained in place for 10 min before removal. Rats recovered in their home cage for 5-7 days until the initial 48 h acclimation period to alcohol.

### Validation

For the site injection and expression validation of viral vectors, rats were anesthetized with isoflurane and transcardially perfused with ice-cold phosphate-buffered saline (PBS), followed by Z-fix zinc formalin fixative (Fisher Scientific, Waltham, MA, USA). Brains were dissected, immersion fixed in Z-fix at 4 °C for 24 h, cryoprotected in 30% sucrose in PBS at 4 °C for 24–48 h, flash frozen in isopentane, and stored at −80 °C. Brains were then sliced on a cryostat into 20 µm thick sections, mounted on SuperFrost Plus slides (Fisher Scientific, Waltham, MA, USA), and stored at −80 °C until use. The CeA was imaged using a Vectra Polaris Imaging system (CLS143455).

For the functional validation of viral vectors, rats were exposed to brief anesthesia (3–5% isoflurane), after which brains were rapidly extracted, placed in ice-cold sucrose solution that contained 206.0 mM sucrose, 2.5 mM KCl, 0.5 mM CaCl_2_, 7.0 mM MgCl_2_, 1.2 mM NaH_2_PO_4_, 26 mM NaHCO_3_, 5.0 mM glucose, and HEPES 5 mM, and coronally sectioned (300 μm) on a Leica VT1000S (Leica Microsystems). After sectioning, slices were incubated in an oxygenated (95% O_2_/5% CO_2_) artificial cerebrospinal fluid (aCSF) solution that contained 130 mM NaCl, 3.5 mM KCl, 1.25 mM NaH_2_PO_4_, 1.5 mM MgSO_4_, 2 mM CaCl_2_, 24 mM NaHCO_3_, and 10 mM glucose for 30 min at 37 °C, followed by 30 min equilibration at room temperature (21-22 °C) as described previously [41, 42]. Electrophysiological recordings were obtained using a Multiclamp 700B amplifier, Digidata 1440 A digitizer, and pClamp 10 software (all from Molecular Devices) at a sampling rate of 20 kHz and low-pass filtered at 10 kHz. CeA neurons that contained mCherry were identified and differentiated from unlabeled neurons using fluorescent optics and brief (<2 s) episcopic illumination. To compare effects of hM4D(Gi) and control virus on spontaneous action potential firing, loose cell-attached recordings were obtained in voltage-clamp mode, and CNO (1 μM) was bath applied for 8-12 min. Four rats were excluded from this analysis because cell-attached recording was unattainable.

### Drugs

Ethyl alcohol (200 proof; 20%) was purchased from Pharmco (Brookfield, CT, USA). CNO was purchased from Cayman Chemical (Ann Arbor, MI, USA), dissolved in 5% DMSO and diluted with 0.9% sterile saline as described previously [43, 44], and administered 1 h before testing. The dose was 2 mg/kg (1 mg/mL, i.p.) based on prior work that showed a reduction of fear conditioning, anxiety-like behavior, and CeA specificity in the same *Crh-*Cre rat line [43, 44].

### Statistical analysis

Behavioral and gene expression data in **Study 1** and **Study 2** were analyzed using analysis of variance (ANOVA), with Stress (unstress vs. stress) and Sex (male *vs*. female) as between-subjects factors. Inhibitory footshock stress data were analyzed using repeated-measures ANOVA, with Shock Day (hit 1 *vs*. hit 2) as the within-subjects factor and Stress as the between-subjects factor. Drinking data in **Study 1** were analyzed using repeated-measures ANOVA, with Drinking across weeks (weeks 1-4) as the within-subjects factor and Stress and Sex as between-subjects factors. Behavioral data in **Study 3** were analyzed using ANOVA, with Virus (AAV8-hSyn-DIO-mCherry *vs*. AAV8-hSyn-DIO-hM4D[Gi]-mCherry), Drug (vehicle *vs*. CNO), and Sex as between-subjects factors. In cases of significant interactions, *post hoc* analyses were conducted using Bonferroni correction adjusted for multiple comparisons. Pearson’s correlation was used to examine relationships between total mean alcohol intake and aggressive and defensive signs as well as peripheral stress hormones. Validation was analyzed using a one sample *t*-test for within-cell percent baseline changes. For all experiments, sample size was pre-determined based on prior work effect sizes using a similar model [31, 39]. Simple randomization for treatment conditions occurred prior to the start of the experiments. Rats were arbitrarily assigned via a numbering system to different treatment groups. All experimenters were blind to the subjects’ treatment condition during testing and sample preparations. All data were analyzed using SPSS 29 software. All graphs were generated using GraphPad Prism 8 software.

## Results

**Fig. 1:**
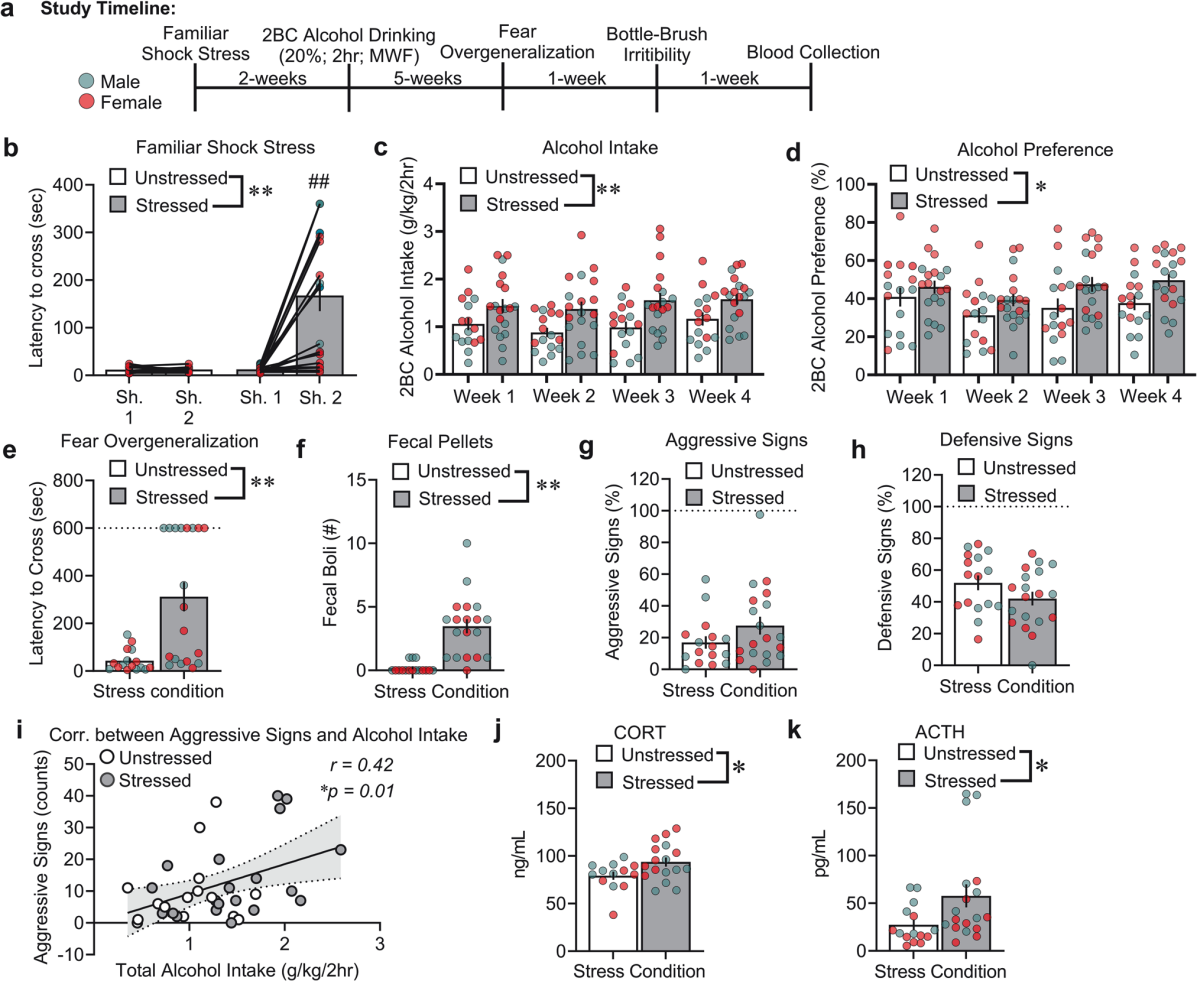
Familiar shock stress increases alcohol drinking, fear overgeneralization, peripheral stress hormones, and alcohol-associated irritability-like behavior. **a** Timeline of experiments in unstressed and stressed male and female rats. **b** Latency to cross (sec) for the “2-hit” familiar shock stress paradigm. **c**, **d** Alcohol intake and preference across the 4 weeks of 2BC alcohol drinking (20%; Monday, Wednesday, Friday; 2 h). **e** Latency to cross (sec) in the fear overgeneralization test. **f** Fecal boli counts (secondary fear response index) during the fear overgeneralization test. **g**, **h** Percent (%) aggressive and defensive signs during the bottle-brush irritability test. **i** Pearson correlation analysis between aggressive signs and total alcohol intake averaged across the 4 weeks of 2BC in unstressed and stressed rats. **j**, **k** Plasma CORT and ACTH levels 1 week after the last behavioral test. ^#^*p* < 0.01, significant difference from shock day; *p < 0.05, **p < 0.01, significant difference from unstressed rats.

**Study 1** employed an established model of familiar context stress and alcohol drinking that exhibits symptomology of comorbid PTSD/AUD. All shocked rats exhibited familiar context avoidance on the second day of shock *vs*. unstressed rats, reflected by a longer latency to cross into the dark side of the familiar shock apparatus (Fig. 1b: Stress: *F*_1,33_ = 18.36, *p* < 0.01, Shock Day × Stress: *F*_1,33_ = 19.36, *p* < 0.001). Stressed rats specifically developed avoidance-like behavior for the shocked paired side on Day 2 *vs*. Day 1 of shock (*p* < 0.001). This effect was not observed in unstressed rats (*p* = 0.998). Beginning 2 weeks after the shock stress procedures, we measured 4 weeks of 2BC intermittent alcohol drinking. These data are expressed as average weekly intake (weeks 1-4) during 2BC alcohol sessions. Familiar shock stress overall increased both alcohol intake and preference *vs*. unstressed controls (Fig. 1c: Stress: *F*_1,31_ = 12.97, *p* = 0.001; Sex: *F*_1,31_ = 11.91, *p* < 0.001; Stress × Sex: *F*_1,31_ = 0.61, *p* = 0.438; Stress × Weeks: *F*_3,93_ = 0.60, *p* = 0.613; Stress × Sex × Weeks: *F*_3,93_ = 0. 96, *p* = 0.415; Fig. 1d: Stress: *F*_1,31_ = 6.06, *p* = 0.02; Sex: *F*_1,31_ = 13.43, *p* < 0.001; Stress × Sex: *F*_1,31_ = 0.006, *p* = 0.939, Stress × Weeks: *F*_3,93_ = 1.09, *p* = 0.357; Stress × Sex × Weeks: *F*_3,93_ = 0.57, *p* = 0.633).

One week after the end of the 2BC drinking procedures, we tested unstressed and stressed rats for fear overgeneralization and irritability-like behavior. Familiar shock stress increased the latency to cross (in seconds) and number of fecal boli (secondary fear response index) *vs*. unstressed control rats in the unfamiliar avoidance apparatus (Fig. 1e: Stress: *F*_1,31_ = 14.85, *p* < 0.01; Sex: *F*_1,31_ = 0.34, *p* = 0.563; Stress × Sex: *F*_1,31_ = 0.33, *p* = 0.569; Fig. 1f: Stress: *F*_1,31_ = 27.31, *p* < 0.001; Sex: *F*_1,31_ = 1.04, *p* = 0.315; Stress × Sex: *F*_1,31_ = 0.17, *p* = 0.677). No significant differences were observed in aggressive or defensive signs (Fig. 1g: Stress: *F*_1,31_ = 2.08, *p* = 0.159; Sex: *F*_1,31_ = 0.84, *p* = 0.365; Stress × Sex: *F*_1,31_ = 0.006, *p* = 0.939; Fig. 1h: Stress: *F*_1,31_ = 2.35, *p* = 0.135; Sex: *F*_1,31_ = 0.01, *p* = 0.906; Stress × Sex: *F*_1,31_ = 0.04, *p* = 0.842). However, the amount of total alcohol intake across the 4 weeks positively correlated with the expression of aggressive signs but not defensive signs (Fig. 1i: *r* = 0.42, 95% confidence interval [CI] = 0.11-0.67, *p* = 0.010; Fig. S2: *r* = −0.19, 95% CI = −0.50-0.15, *p* = 0.255).

We measured enduring changes in peripheral stress hormones in a subset of unstressed *vs*. stressed rats 9 weeks after the initial familiar shock exposure. Familiar shock stress increased plasma CORT and ACTH *vs*. unstressed control rats (Fig. 1j: Stress: *F*_1,26_ = 5.88, *p* = 0.023; Sex: *F*_1,26_ = 0.49, *p* = 0.487; Stress × Sex: *F*_1,26_ = 7.90, *p* = 0.842; Fig. 1k: Stress: *F*_1,29_ = 5.59, *p* = 0.025; Sex: *F*_1,29_ = 8.78, *p* = 0.006; Stress × Sex: *F*_1,29_ = 0.98, *p* = 0.329). No changes were observed in plasma melatonin levels across stress groups (Fig. S3: Stress: *F*_1,25_ = 0.12, *p* = 0.731; Sex: *F*_1,25_ = 10.71, *p* = 0.003; Stress × Sex: *F*_1,25_ = 1.19, *p* = 0.286). Pearson *r* correlation was also performed for plasma CORT and ACTH on total alcohol intake. Plasma CORT or ACTH did not correlate with the levels of total alcohol intake across groups (Fig. S4a: *r* = 0.29, 95% CI = −0.07–0.60, *p* = 0.109; Fig. S4b: *r* = −0.12, 95% CI = −0.45–0.23, *p* = 0.501).

**Fig. 2:**
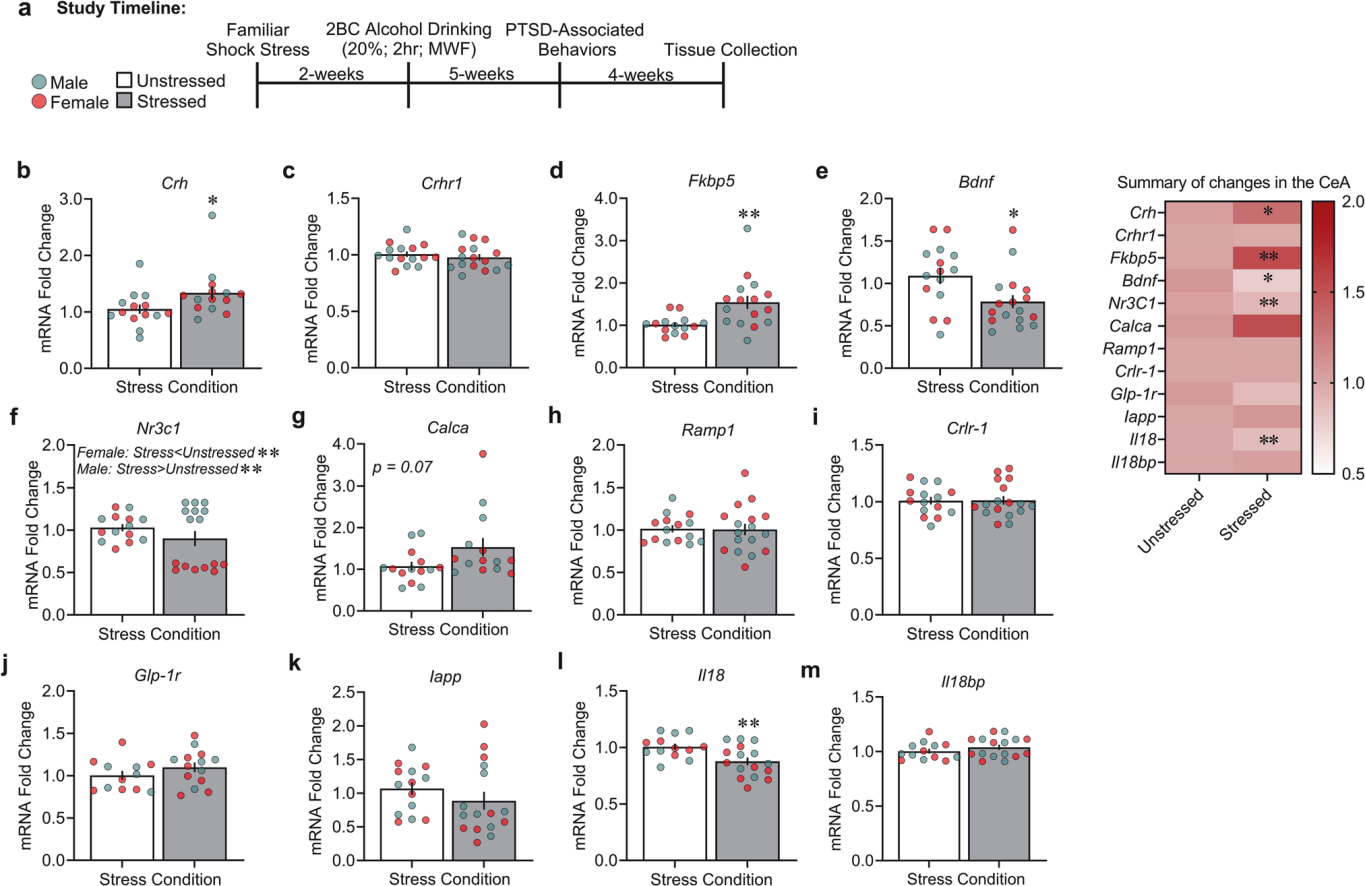
Familiar shock stress and alcohol history disrupt stress- and fear-relevant CeA gene expression in rats. **a** Timeline and tissue collection endpoint. CeA mRNA levels for (**b**). *Crh*, (**c**) *Crhr1*, (**d**) *Fkbp5*, (**e**) *Bdnf*, (**f**) *Nr3c1*, (**g**) *Calca*, (**h**) *Ramp1*, (**i**) C*rlr-1*, (**j**) *Glp-1r*, (**k**) *Iapp*, (**l**) *Il18*, and (**m**) *Il18bp* in unstressed and stressed males and females. *p < 0.05, **p < 0.01, significant difference from unstressed controls.

In **Study 2**, CeA gene expression was analyzed for several stress- and fear-relevant peptides in a separate cohort of rats that were subjected to familiar shock stress and alcohol drinking 11-weeks after their initial shock exposure. Familiar shock stress increased mRNA levels of C*rh* (Fig. 2b: Stress: *F*_1,24_ = 4.46, *p* = 0.048; Sex: *F*_1,24_ = 1.07, *p* = 0.310; Stress × Sex: *F*_1,24_ = 0.24, *p* = 0.623) and *Fkbp5* (Fig. 2d: Stress: *F*_1,26_ = 8.53, *p* = 0.007; Sex: *F*_1,26_ = 0.004, *p* = 0.948; Stress × Sex: *F*_1,26_ = 0.004, *p* = 0.947) and decreased mRNA levels of *Bdnf* (Fig. 2e: Stress: *F*_1,28_ = 6.18, *p* = 0.019; Sex: *F*_1,28_ = 0.53, *p* = 0.472; Stress × Sex: *F*_1,28_ = 0.28, *p* = 0.598) and *Il18* (Fig. 2l: Stress: *F*_1,25_ = 8.58, *p* = 0.007; Sex: *F*_1,25_ = 2.16, *p* = 0.154; Stress × Sex: *F*_1,25_ = 1.66, *p* = 0.209) *vs*. unstressed rats. Furthermore, we observed a significant difference in mRNA levels of *Nr3c1* across males and females (Fig. 2f: Stress: *F*_1,25_ = 8.60, *p* = 0.007; Sex: *F*_1,25_ = 61.59, *p* < 0.001; Stress × Sex: *F*_1,25_ = 52.56, *p* < 0.001). Specifically, familiar shock stress increased *Nr3c1* mRNA levels in males *vs*. their unstressed controls (Fig. 2f; *p* = 0.005), whereas it decreased *Nr3c1* mRNA levels in females *vs*. their respective unstressed controls (Fig. 2f; *p* < 0.001). We observed no significant differences in *Crhr1* or *Calca* gene expression across familiar shock stressed groups (Fig. 2c: Stress: *F*_1,27_ = 0.53, *p* = 0.469; Sex: *F*_1,27_ = 0.45, *p* = 0.507; Stress × Sex: *F*_1,27_ = 0.49, *p* = 0.490; Fig. 2g: Stress: *F*_1,24_ = 3.37, *p* = 0.078; Sex: *F*_1,24_ = 0.02, *p* = 0.871; Stress × Sex: *F*_1,24_ = 0.04, *p* = 0.836).

We also measured the expression of genes that have been more recently associated with fear that might be linked to PTSD/AUD. Familiar shock stress did not significantly alter CeA mRNA levels of *Ramp1 (*Fig. 2h: Stress: *F*_1,28_ = 0.02, *p* = 0.869; Sex: *F*_1,28_ = 1.25, *p* = 0.272; Stress × Sex: *F*_1,28_ = 1.50, *p* = 0.230), C*rlr-1* (Fig. 2i: Stress: *F*_1,28_ = 0.002, *p* = 0.968; Sex: *F*_1,28_ = 2.67, *p* = 0.113; Stress × Sex: *F*_1,28_ = 2.75, *p* = 0.108), *Glp-1r* (Fig. 2j: Stress: *F*_1,27_ = 1.64, *p* = 0.214; Sex: *F*_1,27_ = 0.02, *p* = 0.886; Stress × Sex: *F*_1,27_ = 0.45, *p* = 0.505), *Iapp* (Fig. 2k: Stress: *F*_1,26_ = 1.10, *p* = 0.303; Sex: *F*_1,26_ = 0.21, *p* = 0.644; Stress × Sex: *F*_1,26_ = 0.20, *p* = 0.654), or *Il18bp* (Fig. 2m: Stress: *F*_1,25_ = 1.07, *p* = 0.309; Sex: *F*_1,25_ = 0.002, *p* = 0.965; Stress × Sex: *F*_1,25_ = 0.002, *p* = 0.965).

To examine whether changes in gene expression were altered by stress only (without subsequent alcohol access), we measured a subset of genes in a separate cohort of rats that were only subjected to familiar context stress 48 h after the last shock day. There were no significant differences in CeA mRNA levels of *Crh* (Fig. S5a: Stress: *F*_1,24_ = 0.10, *p* = 0.748; Sex: *F*_1,24_ = 0.02, *p* = 0.872; Stress × Sex: *F*_1,24_ = 0.62, *p* = 0.437), *Crhr1* (Fig. S5b: Stress: *F*_1,25_ = 2.82, *p* = 0.105; Sex: *F*_1,25_ = 1.49, *p* = 0.233; Stress × Sex: *F*_1, 25_ = 1.36, *p* = 0.253), *Fkbp5* (Fig. S5c: Stress: *F*_1,24_ = 2.27, *p* = 0.112; Sex: *F*_1,24_ = 1.04, *p* = 0.317; Stress × Sex: *F*_1,24_ = 0.48, *p* = 0.493), or *Bdnf* (Fig. S5d: Stress: *F*_1,25_ = 0.55, *p* = 0.465; Sex: *F*_1,25_ = 0.07, *p* = 0.793; Stress × Sex: *F*_1,24_ = 0.21, *p* = 0.649) across groups.

**Fig. 3:**
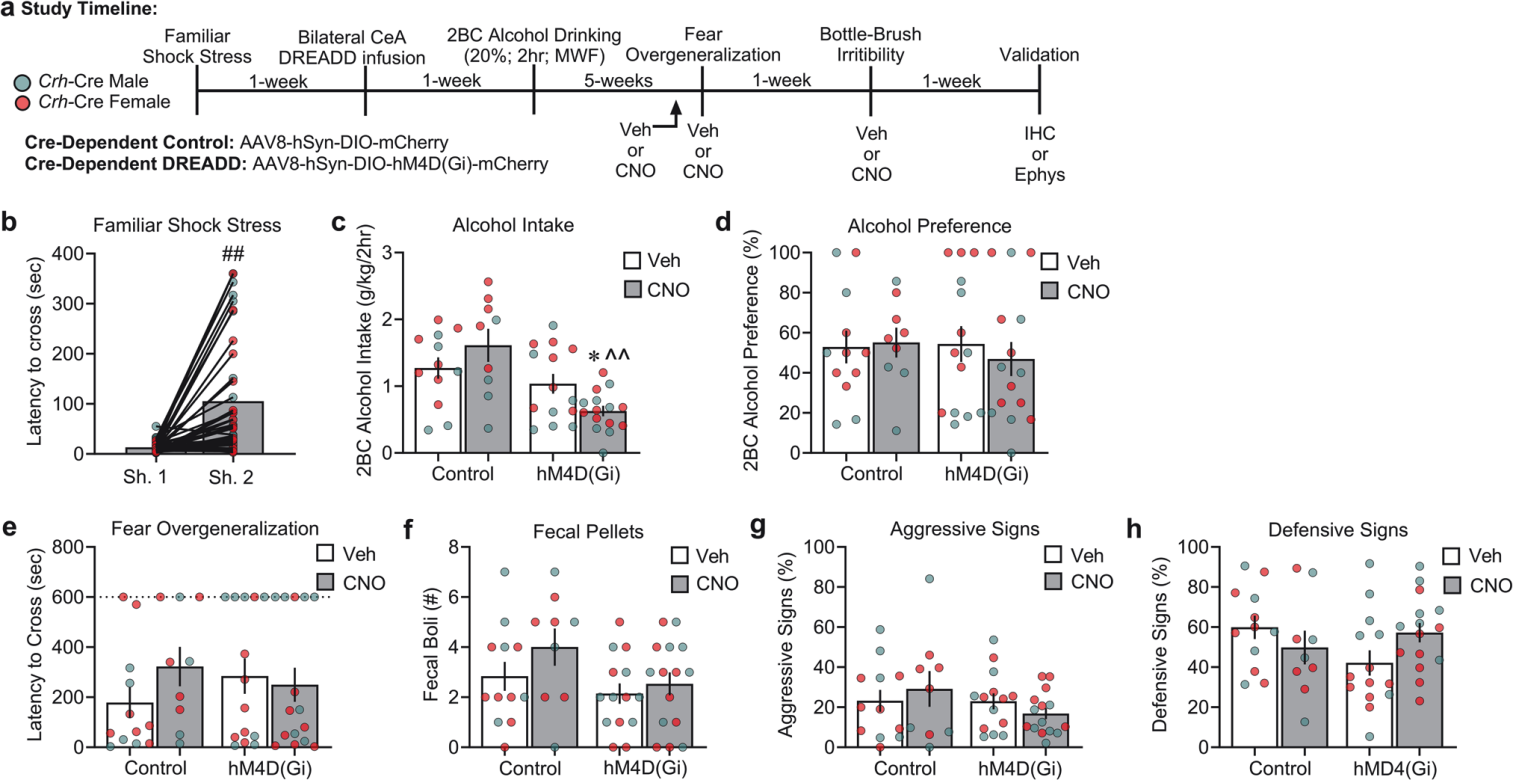
Cre-dependent chemogenetic inhibition of CeA CRF-expressing neurons reduces alcohol intake after familiar shock stress but not fear overgeneralization or irritability-like behavior. **a** Timeline of experiments in CeA viral infused with AAV8-hSyn-DIO-mCherry or AAV8-hSyn-DIO-hM4D(Gi)-mCherry in stressed *Crh*-Cre males and females. **b** Latency to cross (sec) for the “2-hit” familiar shock stress paradigm. **c**, **d** Alcohol intake and preference across the 4 weeks of 2BC alcohol drinking (20%; Monday, Wednesday, Friday; 2 h). **e** Latency to cross (sec) in the fear overgeneralization test. **f** Fecal boli counts (secondary fear response index) during the fear overgeneralization test. **g**, **h** Percent (%) aggressive and defensive signs during the bottle-brush irritability test. ^#^*p* < 0.001, significant difference from shock day 1; *p < 0.05, significant difference from respective vehicle-treated controls; ^^*p* < 0.01, significant difference from CNO (2 mg/kg) mCherry control groups.

In **Study 3**, we used a chemogenetic approach to test whether selectively inactivating CRF-expressing neurons in the CeA reduces comorbid phenotypes of PTSD and alcohol drinking. All *Crh-*Cre rats were stressed and exhibited inhibitory avoidance-like behavior on the second day of shock *vs*. day one (Fig. 3b: Shock Day: *F*_1,48_ = 31.43, *p* < 0.001; Shock Day × Sex: *F*_1,48_ = 4.079, *p* = 0.049; *post hoc*: *p* < 0.001). Furthermore, our analysis revealed a significant difference in alcohol intake across Virus and CNO administration (Fig. 3c: Virus: *F*_1,42_ = 16.14, *p* < 0.001; Drug: *F*_1,42_ = 0.11, *p* = 0.736; Sex: *F*_1,42_ = 10.14, *p* = 0.003; Virus × Drug: *F*_1,42_ = 6.59, *p* = 0.014; Virus × Drug × Sex: *F*_1,42_ = 2.32, *p* = 0.135). Specifically, CNO administration (2 mg/kg) in stressed hDM4(Gi)-expressing rats decreased 2 h alcohol intake *vs*. their respective vehicle-treated controls (*p* = 0.044) and viral vector controls (*p* < 0.001). We observed no significant differences in alcohol preference (Fig. 3d: Virus: *F*_1,42_ = 0.17, *p* = 0.713; Drug: *F*_1,42_ = 0.16, *p* = 0.689; Sex: *F*_1,42_ = 2.48, *p* = 0.123; Virus × Drug: *F*_1,42_ = 0.23, *p* = 0.630; Virus × Drug × Sex: *F*_1,42_ = 0.89, *p* = 0.351). Lastly, no significant differences were observed in water consumption after CNO administration across groups on test day (Fig S6. Virus: *F*_1,42_ = 0.12, *p* = 0.728; Drug: *F*_1,42_ = 0.004, *p* = 0.952; Sex: *F*_1,42_ = 6.14, *p* = 0.017; Virus × Drug × Sex: *F*_1,42_ = 0.54, *p* = 0.465).

One week after the last 2BC alcohol session, we tested for fear overgeneralization and irritability-like behavior following CNO administration (2 mg/kg). Our analysis revealed no significant differences that were produced by CNO administration in either the latency to cross in the fear overgeneralization test (Fig. 3e: Virus: *F*_1,42_ = 0.15, *p* = 0.696; Drug: *F*_1,42_ = 0.69, *p* = 0.411; Sex: *F*_1,42_ = 0.28, *p* = 0.598; Virus × Drug: *F*_1,42_ = 1.46, *p* = 0.233; Virus × Drug × Sex: *F*_1,42_ = 0.22, *p* = 0.636) or fecal boli (Fig. 3f: Virus: *F*_1,42_ = 4.37, *p* = 0.043; Drug: *F*_1,42_ = 1.75, *p* = 0.193; Sex: *F*_1,42_ = 1.08, *p* = 0.304; Virus × Drug: *F*_1,42_ = 0.32, *p* = 0.573; Virus × Drug × Sex: *F*_1,42_ = 1.08, *p* = 0.304). Lastly, we found no significant differences in irritability-like behavior for aggressive signs (Fig. 3g: Virus: *F*_1,42_ = 1.73, *p* = 0.195; Drug: *F*_1,42_ = 0.03, *p* = 0.850; Sex: *F*_1,42_ = 0.11, *p* = 0.738; Virus × Drug: *F*_1,42_ = 1.16, *p* = 0.287; Virus × Drug × Sex: *F*_1,42_ = 0.27, *p* = 0.602). However, we observed significant differences in defensive signs following CNO administration (Fig. 3h: Virus: *F*_1,42_ = 1.73, *p* = 0.195; Drug: *F*_1,42_ = 0.03, *p* = 0.850; Sex: *F*_1,42_ = 0.64, *p* = 0.426; Virus × Drug: *F*_1,42_ = 4.56, *p* = 0.039; Virus × Drug × Sex: *F*_1,42_ = 0.006, *p* = 0.937). Specifically, the Virus × Drug interaction reflected a trend toward an increase in defensive signs after CNO administration (2 mg/kg) in stressed hDM4(Gi)-expressing rats *vs*. their respective vehicle-treated controls (*p* = 0.052).

**Fig. 4:**
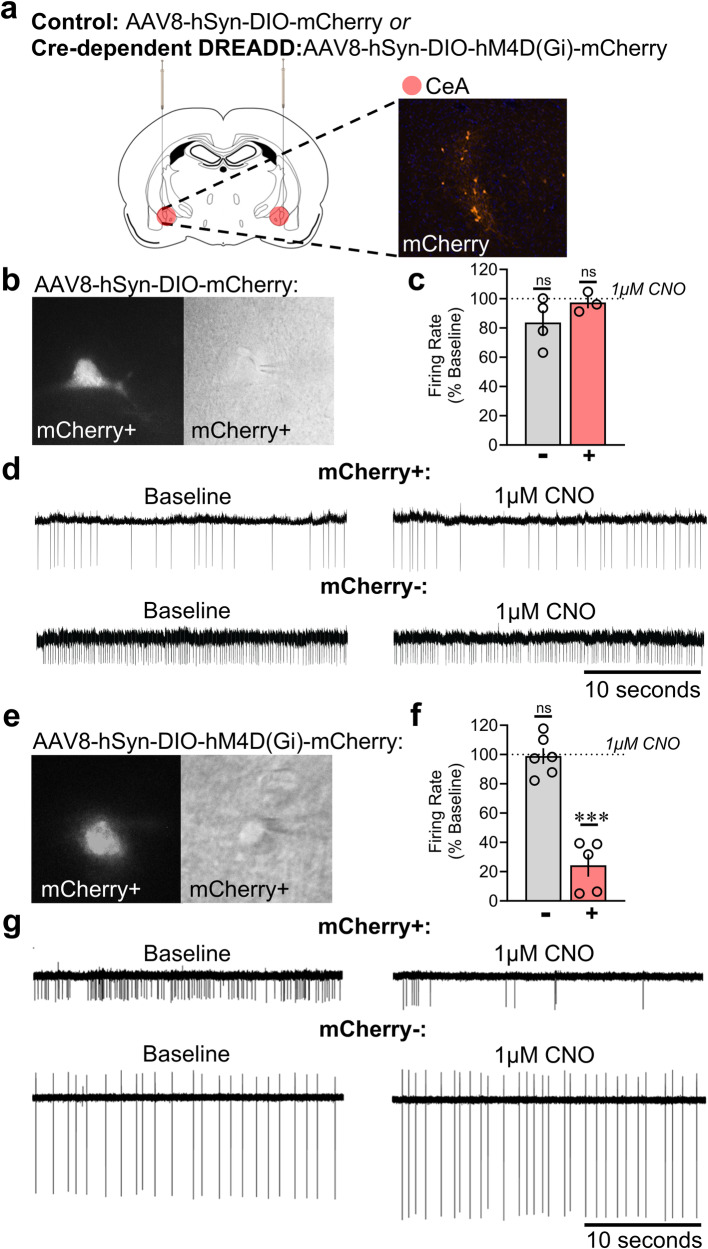
Verification of hM4D(Gi)-DREADD expression and function in the CeA. **a** Site injection and representative expression of mCherry for either control (AAV8-hSyn-DIO-mCherry) or inhibitory DREADD (AAV8-hSyn-DIO-hM4D[Gi]-mCherry) virus transduction in the CeA. **b** Fluorescence image from control vector of an mCherry+ neuron (left), and the same neuron that was patched under differential interference contrast (right). **c** Firing rates from control vector-injected rats were recorded from mCherry+ (*n* = 3) and mCherry− (*n* = 4) CeA neurons. No significant changes in firing rate that were produced by CNO application (1 μM) in either mCherry+ or mCherry- CeA neurons from rats that were injected with control virus. **d** Representative traces of control vector-injected rats before and during CNO application (1 μM). **e** Fluorescence image from inhibitory DREADD vector of an mCherry+ neuron (left), and the same neuron that was patched under differential interference contrast (right). **f** Firing rates of inhibitory DREADD-injected rats were recorded from mCherry+ (*n* = 5) and mCherry− (*n* = 6) CeA neurons. A decrease in firing rate (% Baseline) was produced by CNO application (1 μM) in mCherry+ cells only. **g** Representative traces of DREADD-injected rats before and during CNO application (1 μM).

Viral vectors for control and Cre*-*dependent inhibitory DREADD expression and functional activity were validated at the end of the study in stressed *Crh-*Cre rats. Representative images of viral expression and site injections were measured (Fig. 4a). To assess the functional activity of CRF CeA neurons and specificity of CNO, we recorded mCherry- and mCherry+ neurons in CeA slices from both AAV8-hSyn-DIO-mCherry- and AAV8-hSyn-DIO-hM4D(Gi)-mCherry-injected rats. In AAV8-hSyn-DIO-mCherry-injected (control) rats, CNO (1 μM) did not produce changes in the firing rate of either mCherry- (*t* = 1.98, *p* = 0.145) or mCherry+ (*t* = 0.66, *p* = 0.575) cells (Fig. 4c). Importantly, CNO (1 μM) significantly decreased the firing rate of mCherry+ (*t* = 9.818, *p* = 0.0006) but not mCherry- (*t* = 0.19, *p* = 0.853) CeA neurons from AAV8-hSyn-DIO-hM4D(Gi)-mCherry-injected rats (Fig. 4f).

## Discussion

The present study examined potential CeA gene targets and functional circuit mechanisms that may be involved in comorbid PTSD/AUD in rats. We found similar increases in alcohol drinking, fear overgeneralization, peripheral stress hormone levels, and drinking-related irritability-like behavior after familiar shock stress as previously described [31, 32, 39]. Familiar shock stress and alcohol drinking disrupted the expression of several stress- and fear-relevant genes (including *Crh*) in CeA tissue. Using a chemogenetic approach, we found that the selective inhibition of CRF CeA neurons significantly decreased alcohol intake but not trauma-associated avoidance- or aggressive-like behaviors. Overall, our findings support a role for CeA CRF systems in post-traumatic drinking and add further evidence that other stress targets, including CeA *Fkbp5*, *Bdnf*, and *Nr3c1*, are involved in comorbid PTSD/AUD.

Our findings revealed significant increases in *Crh* and *Fkbp5*, decreases in *Bdnf*, but no change in *Crhr1* gene expression in the CeA that were produced by our comorbid stress and drinking model. We recognize the historical evidence of *Crh* upregulation in the CeA following chronic alcohol withdrawal and stress exposure [45–50]. Our changes in gene expression in the CeA appeared to be driven by the combination of familiar shock stress and alcohol because a subset of stress-only rats, measured 48 h after stress exposure, did not exhibit changes in these genes (see Supplementary Results). However, we note that the lack of changes after 48 h may be attributable to differences in the timing of tissue collection (i.e., 48 h vs. 11 weeks) after the initial shock stress exposure. Importantly, significant increases in CeA *Fkbp5* gene expression were also observed in stressed rats. This finding is consistent with several studies that reported that alcohol withdrawal severity positively correlates with *Fkbp5* levels, PTSD increases DNA methylation for *Fkbp5*, and alcohol administration increases *Fkbp5* mRNA in rats [21–23, 51, 52]. Importantly, recent work from our group showed that inhibiting FKBP5 via broad-acting and selective FKBP5 inhibitors has efficacy in decreasing comorbid PTSD/AUD-like behaviors in rats [39, 53].

CeA *Nr3c1* displayed distinct transcript expression that increased in stressed males and decreased in stressed females *vs*. controls. This finding suggests possible sex differences in the regulation of GR signaling in stressed and alcohol-exposed rats. A recent study from our group found that naive female rats exhibited higher CeA *Nr3c1* gene expression than males, and stress exposure or alcohol may differentially change *Nr3c1* gene transcript levels after a history of stress and alcohol exposure [54]. Parameters in the latter study used in situ hybridization methods, which can account for cell type-selective changes that are not controlled in bulk qPCR gene expression methods. A report that used a high drinking strain of mice (HDID-1) found similar decreases in striatal *Nr3c1* [23]. Alcohol withdrawal also decreases GR mRNA levels in multiple stress- and reward-associated regions [23, 24, 55]. These studies suggest that stress combined with alcohol drinking history may differentially recruit GR systems between males and females. Future studies are needed to assess the sensitivity of GR-targeting compounds and sex differences for the treatment of comorbid PTSD/AUD [30, 39, 54].

We found no significant changes in CeA gene transcript levels for *Ramp1*, *Crlr-1*, *Iapp*, *Glp-1r*, or *Il18bp*. However, transcript levels for *Calca* showed the largest mean-fold change of all studied targets (Fig. 2, summary; *p* = 0.07). This lack of changes does not support their known role for encoding molecules in stress, fear, and drinking behavior [37, 56–58]. Additionally, *Glp-1r* and *Iapp* were shown to play important roles in alcohol drinking and dependence using other rodent models and in other brain areas [36, 59, 60]. Given the trend for altered *Calca* mRNA expression, we cannot exclude changes in CeA expression of this calcitonin-system precursor in our comorbid PTSD/AUD model. Future studies will examine expression of splice-processed transcripts that encode calcitonin or CGRP, given that these systems are linked to threat perception, aversive memories, and alcohol intake. [26, 61]. To our knowledge, these genes have not been explored in the CeA in comorbid PTSD/AUD-like rodent models.

Prior work showed that predator odor stress and chronic alcohol exposure increased CRF expression in the CeA in rodents [14, 48, 50]. We found that *Crh* mRNA was elevated in the CeA in stressed rats. Thus, we examined whether inhibiting CRF-expressing neurons in the CeA reduces behavioral phenotypes of comorbid PTSD/AUD in *Crh-*Cre rats [20, 38]. We found that the chemogenetic inhibition of CRF CeA neurons decreased alcohol intake but not fear overgeneralization or irritability-like behavior. Our results for alcohol consumption are similar to results from a prior study with this same transgenic line, showing that the optogenetic inhibition of CRF CeA neurons decreased the escalation of alcohol intake and somatic signs of withdrawal in alcohol-dependent rats [20]. A surprising finding was that chemogenetic inhibition did not reduce fear overgeneralization or irritability because prior work found that chemogenetic inhibition or caspase3 genetic ablation decreased anxiety-like behavior following immobilization stress in *Crh-*Cre rats [43, 44]. We also acknowledge prior work that showed that the chemogenetic stimulation or inhibition of CeA CRF-expressing neurons did not affect alcohol drinking or negative affective behavior in dependent *Crh*-IRES-Cre mice [48]. We suggest that species differences (*Crh-*Cre rats *vs*. *Crh*-IRES-Cre mice) can yield non-parallel results, particularly for alcohol drinking or differences in the engagement of stress systems (e.g., by shock stress) before alcohol drinking. The lack of changes in trauma-related behaviors might also be mediated by CRF projections outside the CeA, such that the inhibition of CeA CRF neurons alone is insufficient to impact these behaviors.

The neurobiological mechanisms in this report require further assessments. We speculate that comorbid PTSD/AUD are mediated by CeA γ-aminobutyric acid (GABA)-ergic signaling because prior work identified an increase in CeA GABAergic signaling in stressed rats, possibly through CRF, FKBP5 (GR co-chaperone), and *Nr3c1* (which encodes the GR) [31, 39, 62–64]. Indeed, the chemogenetic or optogenetic stimulation of CeA CRF neurons increased GABA transmission (via an increase in spontaneous inhibitory postsynaptic current frequency) in a subset of CeA neurons in *Crh-*Cre rats and *Crh*-IRES-Cre mice, mimicking the increases in GABAergic transmission following familiar stress and alcohol drinking [38, 65]. Another possibility is that familiar shock stress and alcohol drinking may synergistically affect CeA GABAergic transmission, possibly through FKBP5/GR mechanisms, by facilitating the ligand activation of GR and its subsequent translocation into the nucleus [66]. This possibility is supported by work that showed that a GR antagonist decreased CeA GABAergic signaling in alcohol-dependent rats, as well as *Fkbp5* gene expression [63, 67]. Importantly, GR antagonists prevent and reverse the increase in alcohol self-administration that is associated with alcohol dependence and in genetically selected, alcohol-preferring rodent models [24, 25, 55, 68–70]. Future studies are needed to disentangle the mechanisms in stress-mediated alcohol drinking and trauma-induced anxiety.

We note that systemic administration of CNO in DREADD-infused animals can inhibit both local CRF and CRF outside the CeA influencing our behavioral results. Optogenetic inhibition of local CeA CRF neurons prevents escalation of alcohol drinking in *Crh*-Cre rats [20]. Downstream neuronal inhibition of CeA CRF projections to the bed nucleus stria terminalis (BNST) decreases alcohol intake [20]. This inhibition of CRF CeA-BNST pathway is dependent upon the CRF-CRF receptor 1 system with R121919 (CRF_1_ receptor antagonist) blocking the optogenetic inhibition of CRF CeA input neurons [20]. The putative role of CRF outside the extended amygdala structures has also been explored. Excitation of CRF CeA to locus coeruleus (LC) pathway increases anxiety-like behavior, an effect blocked by the administration of antalarmin (CRF_1_ receptor antagonist) [71]. Hypothalamic CRF deficiency in KO mice and inhibition of CRF_1_ in rats decreases anxiety-like behaviors versus controls [72].

In conclusion, we found that familiar shock stress increased phenotypes of comorbid PTSD/AUD, including increases in alcohol drinking, fear overgeneralization, peripheral stress hormone levels, and alcohol-associated irritability-like behavior, recapitulating our prior work and the work of others [31, 32, 39, 73, 74]. Familiar stress and alcohol drinking dysregulate stress and fear-based gene expression in the CeA, providing a region of interest to study the underlying deficits of PTSD/AUD. The selective inhibition of CeA CRF neurons reduced alcohol intake but not fear overgeneralization or irritability-like behavior, suggesting that the modulation of trauma-related behavior may include circuits outside the CeA or other molecular targets. It is essential to examine the expression profiles of other targets such as *Fkbp5* (encodes for GR co-chaperone) or *Nr3c1* (encodes for GR) in the CeA upon CRF inhibition that may provide insight into CRF-related mechanisms. Collectively, these data provide insight into the contribution of CeA CRF and related molecular mechanisms on susceptibility to PTSD/AUD.

## Supplementary information

Supplementary Results

## Data Availability

Data is available upon request.

## References

[1] Zack Ishikawa R, Steere R, Conteh N, Cramer MA, Rao V, Sprich S, et al. Treating PTSD and alcohol use disorder: concurrent cognitive processing therapy and psychopharmacology. J Clin Psychiatry. 2022;84:22ct14636. 36350590 10.4088/JCP.22ct14636

[2] Straus E, Norman SB, Pietrzak RH. Determinants of new-onset alcohol use disorder in U.S. military veterans: Results from the National Health and Resilience in Veterans Study. Addict Behav. 2020;105:106313. 32058235 10.1016/j.addbeh.2020.106313

[3] Smith SM, Goldstein RB, Grant BF. The association between post-traumatic stress disorder and lifetime DSM-5 psychiatric disorders among veterans: Data from the National Epidemiologic Survey on Alcohol and Related Conditions-III (NESARC-III). J Psychiatr Res. 2016;82:16–22. 27455424 10.1016/j.jpsychires.2016.06.022 PMC5026976

[4] Buchholz KR, Bohnert KM, Sripada RK, Rauch SA, Epstein-Ngo QM, Chermack ST. Associations between PTSD and intimate partner and non-partner aggression among substance using veterans in specialty mental health. Addict Behav. 2017;64:194–9. 27636157 10.1016/j.addbeh.2016.08.039 PMC5143184

[5] Brown JM, Williams J, Bray RM, Hourani L. Postdeployment alcohol use, aggression, and post-traumatic stress disorder. Mil Med. 2012;177:1184–90. 23113445 10.7205/milmed-d-11-00119

[6] Norman SB, Haller M, Hamblen JL, Southwick SM, Pietrzak RH. The burden of co-occurring alcohol use disorder and PTSD in U.S. Military veterans: Comorbidities, functioning, and suicidality. Psychol Addict Behav. 2018;32:224–9. 29553778 10.1037/adb0000348

[7] Lebeaut A, Tran JK, Vujanovic AA. Posttraumatic stress, alcohol use severity, and alcohol use motives among firefighters: The role of anxiety sensitivity. Addict Behav. 2020;106:106353. 32087474 10.1016/j.addbeh.2020.106353

[8] Verplaetse TL, McKee SA, Petrakis IL. Pharmacotherapy for co-occurring alcohol use disorder and post-traumatic stress disorder: targeting the opioidergic, noradrenergic, serotonergic, and GABAergic/Glutamatergic systems. Alcohol Res. 2018;39:193–205. 31198658 10.35946/arcr.v39.2.09 PMC6561397

[9] Straus E, Haller M, Lyons RC, Norman SB. Functional and psychiatric correlates of comorbid post-traumatic stress disorder and alcohol use disorder. Alcohol Res. 2018;39:121–9. 31198652 10.35946/arcr.v39.2.03 PMC6561399

[10] Becker HC. Influence of stress associated with chronic alcohol exposure on drinking. Neuropharmacology. 2017;122:115–26. 28431971 10.1016/j.neuropharm.2017.04.028 PMC5497303

[11] Becker HC. Effects of alcohol dependence and withdrawal on stress responsiveness and alcohol consumption. Alcohol Res. 2012;34:448–58. 23584111 10.35946/arcr.v34.4.09 PMC3860383

[12] Stephens MA, Wand G. Stress and the HPA axis: role of glucocorticoids in alcohol dependence. Alcohol Res. 2012;34:468–83. 23584113 10.35946/arcr.v34.4.11 PMC3860380

[13] Gilpin NW, Herman MA, Roberto M. The central amygdala as an integrative hub for anxiety and alcohol use disorders. Biol Psychiatry. 2015;77:859–69. 25433901 10.1016/j.biopsych.2014.09.008 PMC4398579

[14] Roberto M, Spierling SR, Kirson D, Zorrilla EP. Corticotropin-releasing factor (CRF) and addictive behaviors. Int Rev Neurobiol. 2017;136:5–51. 29056155 10.1016/bs.irn.2017.06.004 PMC6155477

[15] Merlo Pich E, Lorang M, Yeganeh M, Rodriguez de Fonseca F, Raber J, Koob GF, et al. Increase of extracellular corticotropin-releasing factor-like immunoreactivity levels in the amygdala of awake rats during restraint stress and ethanol withdrawal as measured by microdialysis. J Neurosci. 1995;15:5439–47. 7643193 10.1523/JNEUROSCI.15-08-05439.1995 PMC6577636

[16] Sabino V, Cottone P, Koob GF, Steardo L, Lee MJ, Rice KC, et al. Dissociation between opioid and CRF1 antagonist sensitive drinking in Sardinian alcohol-preferring rats. Psychopharmacol (Berl). 2006;189:175–86. 10.1007/s00213-006-0546-5 17047935

[17] Funk CK, Zorrilla EP, Lee MJ, Rice KC, Koob GF. Corticotropin-releasing factor 1 antagonists selectively reduce ethanol self-administration in ethanol-dependent rats. Biol Psychiatry. 2007;61:78–86. 16876134 10.1016/j.biopsych.2006.03.063 PMC2741496

[18] Chu K, Koob GF, Cole M, Zorrilla EP, Roberts AJ. Dependence-induced increases in ethanol self-administration in mice are blocked by the CRF1 receptor antagonist antalarmin and by CRF1 receptor knockout. Pharm Biochem Behav. 2007;86:813–21. 10.1016/j.pbb.2007.03.009 PMC2170886 17482248

[19] Huang MM, Overstreet DH, Knapp DJ, Angel R, Wills TA, Navarro M, et al. Corticotropin-releasing factor (CRF) sensitization of ethanol withdrawal-induced anxiety-like behavior is brain site specific and mediated by CRF-1 receptors: relation to stress-induced sensitization. J Pharm Exp Ther. 2010;332:298–307. 10.1124/jpet.109.159186 PMC2802475 19843974

[20] de Guglielmo G, Kallupi M, Pomrenze MB, Crawford E, Simpson S, Schweitzer P, et al. Inactivation of a CRF-dependent amygdalofugal pathway reverses addiction-like behaviors in alcohol-dependent rats. Nat Commun. 2019;10:1238. 30886240 10.1038/s41467-019-09183-0 PMC6423296

[21] Nylander I, Todkar A, Granholm L, Vrettou M, Bendre M, Boon W, et al. Evidence for a link between Fkbp5/FKBP5, early life social relations and alcohol drinking in young adult rats and humans. Mol Neurobiol. 2017;54:6225–34. 27709495 10.1007/s12035-016-0157-z PMC5583263

[22] Lieberman R, Armeli S, Scott DM, Kranzler HR, Tennen H, Covault J. FKBP5 genotype interacts with early life trauma to predict heavy drinking in college students. Am J Med Genet B Neuropsychiatr Genet. 2016;171:879–87. 27196697 10.1002/ajmg.b.32460 PMC5045724

[23] Savarese AM, Grigsby KB, Jensen BE, Borrego MB, Finn DA, Crabbe JC, et al. Corticosterone levels and glucocorticoid receptor gene expression in high drinking in the dark mice and their heterogeneous stock (HS/NPT) founder line. Front Behav Neurosci. 2022;16:821859. 35645743 10.3389/fnbeh.2022.821859 PMC9135139

[24] Vendruscolo LF, Barbier E, Schlosburg JE, Misra KK, Whitfield TW Jr, Logrip ML, et al. Corticosteroid-dependent plasticity mediates compulsive alcohol drinking in rats. J Neurosci. 2012;32:7563–71. 22649234 10.1523/JNEUROSCI.0069-12.2012 PMC3375621

[25] Vendruscolo LF, Estey D, Goodell V, Macshane LG, Logrip ML, Schlosburg JE, et al. Glucocorticoid receptor antagonism decreases alcohol seeking in alcohol-dependent individuals. J Clin Invest. 2015;125:3193–7. 26121746 10.1172/JCI79828 PMC4563748

[26] Brancato A, Castelli V, Cannizzaro C, Tringali G. Adolescent binge-like alcohol exposure dysregulates NPY and CGRP in rats: Behavioural and immunochemical evidence. Prog Neuropsychopharmacol Biol Psychiatry. 2023;123:110699. 36565980 10.1016/j.pnpbp.2022.110699

[27] Neugebauer V, Mazzitelli M, Cragg B, Ji G, Navratilova E, Porreca F. Amygdala, neuropeptides, and chronic pain-related affective behaviors. Neuropharmacology. 2020;170:108052. 32188569 10.1016/j.neuropharm.2020.108052 PMC7214122

[28] Li X, Kovacs EJ, Schwacha MG, Chaudry IH, Choudhry MA. Acute alcohol intoxication increases interleukin-18-mediated neutrophil infiltration and lung inflammation following burn injury in rats. Am J Physiol Lung Cell Mol Physiol. 2007;292:L1193–1201. 17220368 10.1152/ajplung.00408.2006

[29] Sugama S, Conti B. Interleukin-18 and stress. Brain Res Rev. 2008;58:85–95. 18295340 10.1016/j.brainresrev.2007.11.003

[30] Borgonetti V, Cruz B, Vozella V, Khom S, Steinman MQ, Bullard R, et al. IL-18 signaling in the rat central amygdala is disrupted in a comorbid model of post-traumatic stress and alcohol use disorders. Cells. 2023;12:1943. 10.3390/cells12151943 PMC10416956 37566022

[31] Steinman MQ, Kirson D, Wolfe SA, Khom S, D’Ambrosio SR, Spierling Bagsic SR, et al. Importance of sex and trauma context on circulating cytokines and amygdalar GABAergic signaling in a comorbid model of posttraumatic stress and alcohol use disorders. Mol Psychiatry. 2021;26:3093–107. 33087855 10.1038/s41380-020-00920-2 PMC8058115

[32] Kirson D, Steinman MQ, Wolfe SA, Spierling Bagsic SR, Bajo M, Sureshchandra S, et al. Sex and context differences in the effects of trauma on comorbid alcohol use and post-traumatic stress phenotypes in actively drinking rats. J Neurosci Res. 2021;99:3354–72. 34687080 10.1002/jnr.24972 PMC8712392

[33] Kosten TA, Huang W, Nielsen DA. Sex and litter effects on anxiety and DNA methylation levels of stress and neurotrophin genes in adolescent rats. Dev Psychobiol. 2014;56:392–406. 23460384 10.1002/dev.21106

[34] Klausen MK, Thomsen M, Wortwein G, Fink-Jensen A. The role of glucagon-like peptide 1 (GLP-1) in addictive disorders. Br J Pharm. 2022;179:625–41. 10.1111/bph.15677 PMC8820218 34532853

[35] Sink KS, Walker DL, Yang Y, Davis M. Calcitonin gene-related peptide in the bed nucleus of the stria terminalis produces an anxiety-like pattern of behavior and increases neural activation in anxiety-related structures. J Neurosci. 2011;31:1802–10. 21289190 10.1523/JNEUROSCI.5274-10.2011 PMC3088995

[36] Kalafateli AL, Vallof D, Jerlhag E. Activation of amylin receptors attenuates alcohol-mediated behaviours in rodents. Addict Biol. 2019;24:388–402. 29405517 10.1111/adb.12603 PMC6585842

[37] Kalafateli AL, Vestlund J, Raun K, Egecioglu E, Jerlhag E. Effects of a selective long-acting amylin receptor agonist on alcohol consumption, food intake and body weight in male and female rats. Addict Biol. 2021;26:e12910. 32383257 10.1111/adb.12910

[38] Pomrenze MB, Millan EZ, Hopf FW, Keiflin R, Maiya R, Blasio A, et al. A transgenic rat for investigating the anatomy and function of corticotrophin releasing factor circuits. Front Neurosci. 2015;9:487. 26733798 10.3389/fnins.2015.00487 PMC4689854

[39] Cruz B, Vozella V, Carper BA, Xu JC, Kirson D, Hirsch S, et al. FKBP5 inhibitors modulate alcohol drinking and trauma-related behaviors in a model of comorbid post-traumatic stress and alcohol use disorder. Neuropsychopharmacology. 2022. 10.1038/s41386-022-01497-w PMC10267127 36396784

[40] Flores-Ramirez FJ, Varodayan FP, Patel RR, Illenberger JM, Di Ottavio F, Roberto M, et al. Blockade of orexin receptors in the infralimbic cortex prevents stress-induced reinstatement of alcohol-seeking behaviour in alcohol-dependent rats. Br J Pharm. 2023;180:1500–15. 10.1111/bph.16015 PMC10577928 36537731

[41] Khom S, Wolfe SA, Patel RR, Kirson D, Hedges DM, Varodayan FP, et al. Alcohol dependence and withdrawal impair serotonergic regulation of GABA transmission in the rat central nucleus of the amygdala. J Neurosci. 2020;40:6842–53. 32769108 10.1523/JNEUROSCI.0733-20.2020 PMC7470924

[42] Varodayan FP, Patel RR, Matzeu A, Wolfe SA, Curley DE, Khom S, et al. The amygdala noradrenergic system is compromised with alcohol use disorder. Biol Psychiatry. 2022;91:1008–18. 35430085 10.1016/j.biopsych.2022.02.006 PMC9167785

[43] Pomrenze MB, Tovar-Diaz J, Blasio A, Maiya R, Giovanetti SM, Lei K, et al. A corticotropin releasing factor network in the extended amygdala for anxiety. J Neurosci. 2019;39:1030–43. 30530860 10.1523/JNEUROSCI.2143-18.2018 PMC6363927

[44] Pomrenze MB, Giovanetti SM, Maiya R, Gordon AG, Kreeger LJ, Messing RO. Dissecting the roles of GABA and neuropeptides from rat central amygdala CRF neurons in anxiety and fear learning. Cell Rep. 2019;29:13–21.e14. 31577943 10.1016/j.celrep.2019.08.083 PMC6879108

[45] Roberto M, Cruz MT, Gilpin NW, Sabino V, Schweitzer P, Bajo M, et al. Corticotropin releasing factor-induced amygdala gamma-aminobutyric Acid release plays a key role in alcohol dependence. Biol Psychiatry. 2010;67:831–9. 20060104 10.1016/j.biopsych.2009.11.007 PMC2883449

[46] Sommer WH, Rimondini R, Hansson AC, Hipskind PA, Gehlert DR, Barr CS, et al. Upregulation of voluntary alcohol intake, behavioral sensitivity to stress, and amygdala crhr1 expression following a history of dependence. Biol Psychiatry. 2008;63:139–45. 17585886 10.1016/j.biopsych.2007.01.010

[47] Zorrilla EP, Valdez GR, Weiss F. Changes in levels of regional CRF-like-immunoreactivity and plasma corticosterone during protracted drug withdrawal in dependent rats. Psychopharmacol (Berl). 2001;158:374–81. 10.1007/s002130100773 11797058

[48] Kreifeldt M, Herman MA, Sidhu H, Okhuarobo A, Macedo GC, Shahryari R, et al. Central amygdala corticotropin-releasing factor neurons promote hyponeophagia but do not control alcohol drinking in mice. Mol Psychiatry. 2022;27:2502–13. 35264727 10.1038/s41380-022-01496-9 PMC9149056

[49] Ciccocioppo R, de Guglielmo G, Hansson AC, Ubaldi M, Kallupi M, Cruz MT, et al. Restraint stress alters nociceptin/orphanin FQ and CRF systems in the rat central amygdala: significance for anxiety-like behaviors. J Neurosci. 2014;34:363–72. 24403138 10.1523/JNEUROSCI.2400-13.2014 PMC3870926

[50] Weera MM, Schreiber AL, Avegno EM, Gilpin NW. The role of central amygdala corticotropin-releasing factor in predator odor stress-induced avoidance behavior and escalated alcohol drinking in rats. Neuropharmacology. 2020;166:107979. 32028150 10.1016/j.neuropharm.2020.107979 PMC7442223

[51] Huang MC, Schwandt ML, Chester JA, Kirchhoff AM, Kao CF, Liang T, et al. FKBP5 moderates alcohol withdrawal severity: human genetic association and functional validation in knockout mice. Neuropsychopharmacology. 2014;39:2029–38. 24603855 10.1038/npp.2014.55 PMC4059914

[52] Kang JI, Kim TY, Choi JH, So HS, Kim SJ. Allele-specific DNA methylation level of FKBP5 is associated with post-traumatic stress disorder. Psychoneuroendocrinology. 2019;103:1–7. 30605803 10.1016/j.psyneuen.2018.12.226

[53] Sakoulas EM, Bertotto LB, Logrip ML, Lin K, Pimentel AE, Cruz B, et al. Benztropine reduces reacquisition of alcohol self-administration in rats with stress history: role of FKBP5. Federation Am Soc Exp Biol. 2022;36:L8036.

[54] Khom S, Borgonetti V, Vozella V, Kirson D, Rodriguez L, Gandhi P, et al. Glucocorticoid receptors regulate central amygdala GABAergic synapses in Marchigian-Sardinian alcohol-preferring rats. Neurobiol Stress. 2023;25:100547. 37547774 10.1016/j.ynstr.2023.100547 PMC10401345

[55] Somkuwar SS, Vendruscolo LF, Fannon MJ, Schmeichel BE, Nguyen TB, Guevara J, et al. Abstinence from prolonged ethanol exposure affects plasma corticosterone, glucocorticoid receptor signaling and stress-related behaviors. Psychoneuroendocrinology. 2017;84:17–31. 28647675 10.1016/j.psyneuen.2017.06.006 PMC5557646

[56] Hwang BH, Kunkler PE, Lumeng L, Li TK. Calcitonin gene-related peptide (CGRP) content and CGRP receptor binding sites in discrete forebrain regions of alcohol-preferring vs. -nonpreferring rats, and high alcohol-drinking vs. low alcohol-drinking rats. Brain Res. 1995;690:249–53. 8535845 10.1016/0006-8993(95)00636-5

[57] Palmiter RD. The parabrachial nucleus: CGRp neurons function as a general alarm. Trends Neurosci. 2018;41:280–93. 29703377 10.1016/j.tins.2018.03.007 PMC5929477

[58] Wu X, Zhang JT, Liu J, Yang S, Chen T, Chen JG, et al. Calcitonin gene-related peptide erases the fear memory and facilitates long-term potentiation in the central nucleus of the amygdala in rats. J Neurochem. 2015;135:787–98. 26179152 10.1111/jnc.13246

[59] Shirazi RH, Dickson SL, Skibicka KP. Gut peptide GLP-1 and its analogue, Exendin-4, decrease alcohol intake and reward. PLoS One. 2013;8:e61965. 23613987 10.1371/journal.pone.0061965 PMC3628574

[60] Suchankova P, Yan J, Schwandt ML, Stangl BL, Caparelli EC, Momenan R, et al. The glucagon-like peptide-1 receptor as a potential treatment target in alcohol use disorder: evidence from human genetic association studies and a mouse model of alcohol dependence. Transl Psychiatry. 2015;5:e583. 26080318 10.1038/tp.2015.68 PMC4490279

[61] Kang SJ, Liu S, Ye M, Kim DI, Pao GM, Copits BA, et al. A central alarm system that gates multi-sensory innate threat cues to the amygdala. Cell Rep. 2022;40:111222. 35977501 10.1016/j.celrep.2022.111222 PMC9420642

[62] Scharf SH, Liebl C, Binder EB, Schmidt MV, Muller MB. Expression and regulation of the Fkbp5 gene in the adult mouse brain. PLoS One. 2011;6:e16883. 21347384 10.1371/journal.pone.0016883 PMC3036725

[63] Khom S, Rodriguez L, Gandhi P, Kirson D, Bajo M, Oleata CS, et al. Alcohol dependence and withdrawal increase sensitivity of central amygdalar GABAergic synapses to the glucocorticoid receptor antagonist mifepristone in male rats. Neurobiol Dis. 2022;164:105610. 34995754 10.1016/j.nbd.2022.105610 PMC9301881

[64] Papilloud A, Veenit V, Tzanoulinou S, Riccio O, Zanoletti O, Guillot de Suduiraut I, et al. Peripubertal stress-induced heightened aggression: modulation of the glucocorticoid receptor in the central amygdala and normalization by mifepristone treatment. Neuropsychopharmacology. 2019;44:674–82. 29941978 10.1038/s41386-018-0110-0 PMC6372583

[65] Sanford CA, Soden ME, Baird MA, Miller SM, Schulkin J, Palmiter RD, et al. A Central Amygdala CRF Circuit Facilitates Learning about Weak Threats. Neuron. 2017;93:164–78. 28017470 10.1016/j.neuron.2016.11.034 PMC5217711

[66] Zannas AS, Wiechmann T, Gassen NC, Binder EB. Gene-stress-epigenetic regulation of FKBP5: clinical and translational implications. Neuropsychopharmacology. 2016;41:261–74. 26250598 10.1038/npp.2015.235 PMC4677131

[67] Bali U, Phillips T, Hunt H, Unitt J. FKBP5 mRNA expression is a biomarker for GR antagonism. J Clin Endocrinol Metab. 2016;101:4305–12. 27459525 10.1210/jc.2016-1624

[68] Savarese AM, Ozburn AR, Metten P, Schlumbohm JP, Hack WR, LeMoine K, et al. Targeting the glucocorticoid receptor reduces binge-like drinking in high drinking in the dark (HDID-1) mice. Alcohol Clin Exp Res. 2020;44:1025–36. 32154593 10.1111/acer.14318 PMC7211124

[69] Benvenuti F, Cannella N, Stopponi S, Soverchia L, Ubaldi M, Lunerti V, et al. Effect of glucocorticoid receptor antagonism on alcohol self-administration in genetically-selected marchigian sardinian alcohol-preferring and non-preferring wistar rats. Int J Mol Sci. 2021;22:4184. 33920737 10.3390/ijms22084184 PMC8073469

[70] McGinn MA, Tunstall BJ, Schlosburg JE, Gregory-Flores A, George O, de Guglielmo G, et al. Glucocorticoid receptor modulators decrease alcohol self-administration in male rats. Neuropharmacology. 2021;188:108510. 33647278 10.1016/j.neuropharm.2021.108510 PMC8099171

[71] Paretkar T, Dimitrov E. The central amygdala corticotropin-releasing hormone (CRH) neurons modulation of anxiety-like behavior and hippocampus-dependent memory in mice. Neuroscience. 2018;390:187–97. 30170157 10.1016/j.neuroscience.2018.08.019 PMC6168391

[72] Zhang R, Asai M, Mahoney CE, Joachim M, Shen Y, Gunner G, et al. Loss of hypothalamic corticotropin-releasing hormone markedly reduces anxiety behaviors in mice. Mol Psychiatry. 2017;22:733–44. 27595593 10.1038/mp.2016.136 PMC5339066

[73] Gomez JL, Luine VN. Female rats exposed to stress and alcohol show impaired memory and increased depressive-like behaviors. Physiol Behav. 2014;123:47–54. 24096191 10.1016/j.physbeh.2013.09.009 PMC3881970

[74] Alavi M, Ryabinin AE, Helms ML, Nipper MA, Devaud LL, Finn DA. Sensitivity and resilience to predator stress-enhanced ethanol drinking is associated with sex-dependent differences in stress-regulating systems. Front Behav Neurosci. 2022;16:834880. 35645747 10.3389/fnbeh.2022.834880 PMC9132579

