# Supplementary material for: Chemogenetic inhibition of central amygdala CRF-expressing neurons decreases alcohol intake but not trauma-related behaviors in a rat model of post-traumatic stress and alcohol use disorder
Source: Mol Psychiatry. 2024 Mar 21;29(9):2611–21. doi: 10.1038/s41380-024-02514-8 (PMC11415545; doi:10.1038/s41380-024-02514-8)
Supplement: Supplementary file 1 — Supplementary Results [file 41380_2024_2514_MOESM1_ESM.docx]

**Supplementary Results**

**Figure S1.**

**Figure S1.** Non-detectable and detectable blood alcohol levels (BALs) expressed alcohol intake and preference of all rats. Blood alcohol levels were assessed after the 30 min into the rats’ 2 h drinking period on the last week of the 2BC regimen (Day 11). Those with detectable BALs exhibited significantly higher 2BC intake and preference compared with intake and preference for non-detectable levels (**Fig. S1a**: *t*_80_ = 3.594, *p* = 0.0006; **Fig. S1b**: *t*_80_ = 4.652, *p* < 0.0001). The data are expressed as the mean ± SEM. ***p* ≤ 0.01, ****p* ≤ 0.001, significant difference from non-detectable group.

**Figure S2.**

**Figure S2.** Pearson *r* correlation between defensive signs and total alcohol intake across the 4 weeks of drinking. Expression of defensive signs did not correlate with the levels of total alcohol intake in stress groups (*r* = -0.19, 95% CI = -0.50- 0.15, p = 0.255).

**Figure S3.**

**Figure S3.** Plasma melatonin levels assessed after familiar shock stress and alcohol history. This graph reflects pg/ml levels of melatonin in unstressed (white bars) and stressed (grey bars) male (blue) and female (red) rats. No changes were observed in plasma melatonin levels across stress groups (Stress: *F*_1,25_ = 0.121, *p* = 0.731; Sex: *F*_1,25_ = 10.71, *p* = 0.003; Stress × Sex: *F*_1,25_ = 1.190, *p* = 0.286).

**Figure S4.**

**Figure S4.** Pearson *r* correlation between CORT or ACTH and total alcohol intake across 4 weeks of drinking. Expression of plasma CORT or ACTH did not correlate with the levels of total alcohol intake across groups (**Fig. S4a:** *r* = 0.29, 95% CI = -0.070-0.60, *p* = 0.109; **Fig. S4b:** *r* = -0.12, 95% CI = -0.45-0.23, *p* = 0.501).

**Figure S5.**

**Figure S5.** CeA mRNA levels of *Crh*, *Crhr1*, *Fkbp5*, and *Bdnf* measured 48 h after the last familiar shock exposure. The graph reflects unstressed (white bars) and stressed (grey bars) male (blue) and female (red) rats. No changes were observed in these CeA genes measured 48 h after the last shock stress procedure (**Fig. S5a**: Stress: *F*_1,24_ = 0.106, *p* = 0.748; Sex: *F*_1,24_ = 0.026, *p* = 0.872; Stress × Sex: *F*_1,24_ = 0.625, *p* = 0.437; **Fig. S5b**: Stress: *F*_1,25_ = 2.82, *p* = 0.105; Sex: *F*_1,25_ = 1.492, *p* = 0.233; Stress × Sex: *F*_1,25_ = 1.368, *p* = 0.253; **Fig. S5c**: Stress: *F*_1,24_ = 2.272, *p* = 0.112; Sex: *F*_1,24_ = 1.045, *p* = 0.317; Stress × Sex: *F*_1,24_ = 0.485, *p* = 0.493; **Fig. S5d**: Stress: *F*_1,25_ = 0.551, *p* = 0.465; Sex: *F*_1,25_ = 0.070, *p* = 0.793; Stress × Sex: *F*_1,24_ = 0.213, *p* = 0.649).

**Figure S6.**

**Figure S6.** Water consumption after CNO administration on 2BC drinking test day. This graph reflects vehicle (white bars) and CNO (grey bars; 2 mg/kg) treated male (blue) and female (red) rats infused with AAV8-hSyn-DIO-mCherry or AAV8-hSyn-DIO-hM4D(Gi)-mCherry. No significant changes were observed in water consumption by CNO administration across groups on test day (Sex: *F*_1,42_ = 6.144, *p* = 0.017; Virus: *F*_1,42_ = 0.123, *p* = 0.728; Drug: *F*_1,42_ = 0.004, *p* = 0.952; Sex × Virus × Drug: *F*_1,42_ = 0.544, *p* = 0.465).
